# Periodontal Diseases as Putative Risk Factors for Head and Neck Cancer: Systematic Review and Meta-Analysis

**DOI:** 10.3390/cancers12071893

**Published:** 2020-07-14

**Authors:** Divya Gopinath, Rohit Kunnath Menon, Sajesh K. Veettil, Michael George Botelho, Newell W. Johnson

**Affiliations:** 1 Oral Diagnostics and Surgical Sciences, International Medical University, Kuala Lumpur 57000, Malaysia; 2 Clinical Dentistry, International Medical University, Kuala Lumpur 57000, Malaysia; rohitkunnath@imu.edu.my; 3 School of Pharmacy, International Medical University, Kuala Lumpur 57000, Malaysia; sajesh.vijay@gmail.com; 4 Department of Prosthodontics, Faculty of Dentistry, The University of Hong Kong, Hong Kong; n.johnson@griffith.edu.au; 5 Menzies Health Institute Queensland and School of Dentistry and Oral Health, Griffith University, Gold Coast, QLD 4222, Australia; 6 Faculty of Dentistry, Oral and Craniofacial Sciences, King’s College London, London WC2R 2LS, UK

**Keywords:** head and neck cancer, oral cancer, periodontitis, periodontal disease, periodontium, association

## Abstract

Whether “periodontal disease” can be considered as an independent risk factor for head and neck cancer (HNC) remains controversial. The aim of the current meta-analysis was to quantitatively assess this relationship in order to determine whether this represents a true risk factor, with implications for cancer prevention and management. PubMed, Scopus, and Embase databases were systematically searched. Selective studies were reviewed, and meta-analysis was performed to estimate the pooled odds ratio (OR) with 95% confidence intervals (CIs) on eligible studies using a random effects model. In total, 21 eligible observational studies (4 cohorts and 17 case-controls) were identified for qualitative synthesis after a review of 1051 articles. Significant heterogeneity could be identified in measures utilized for reporting of periodontal disease. Meta-analysis performed on nine studies that employed objective measures for reporting periodontal disease demonstrated a significant association between periodontal disease and HNC [OR 3.17, 95% CI, 1.78–5.64]. A diseased periodontium represents an independent risk marker, and a putative risk factor, for HNC. Prospective studies with standardized measures of periodontal disease severity and extent, integrated with microbiological and host susceptibility facets, are needed to elucidate the mechanisms of this positive association and whether treatment of the former influences the incidence and outcomes for HNC.

## 1. Introduction

Periodontal health is defined by the absence of clinically detectable inflammation of the gingivae or periodontal membrane, without periodontal pockets or ongoing loss of attachment of the teeth to the alveolar bone [1]. “Periodontal disease” is a broad term covering diseased conditions of the periodontium, encompassing gingivitis, periodontitis, and often other conditions [1,2,3,4]. Gingivitis is a non-specific inflammatory condition as a result of sustained plaque biofilm accumulation at, and apical to, the gingival margin [1]. Gingivitis is currently considered as a genuine risk factor for, and pre-requisite to, the development of periodontitis, even though not all gingivitis may lead to periodontitis [1,3]. Periodontitis, on the other hand, is a progressive disease, which evolves over time as a result of biofilm accumulation at the gingival margins of teeth, coupled to an inability of the host to eliminate the putatively pathogenic micro flora, which can lead to subgingival microbial colonization, sustained inflammation, and bystander tissue destruction, leading to periodontal pockets because of loss of connective attachment and associated bone loss [5,6].

Periodontal diseases have been strongly linked with numerous systemic conditions, including diabetes mellitus, cardiovascular diseases, poor pregnancy outcomes, and pulmonary, renal, and immunologic disorders [6]. Whilst these associations have been investigated for several decades, there is a recent upsurge on publications linking periodontal disease and cancer risk, both in regard to local malignancies and those at distant sites, with chronic inflammation and immune dysfunction serving as the chief foci for logical interpretation. The periodontal pathogens *Fusobacterium nucleatum* and *Porphyromonas gingivalis* are often isolated from malignant neoplasms of the oral cavity [7,8] as well as other cancers [9], intensifying the biological plausibility of a causative link. Indirect mechanisms re-enforcing links between periodontitis and cancer encompass the role of inflammatory mediators in cancer progression, as a chronically inflamed periodontium continuously releases such factors into the circulation in substantial quantities [10].

The term head and neck cancer includes all malignancies of the upper aerodigestive tract (UADT), the majority of which are of mucosal origin, arising from the paranasal sinuses; nasal cavity; oral cavity; naso-, oro-, and hypo-pharynx; and larynx [11]. Several of these sites have contiguity or proximity to the oral cavity and may be influenced by inflammatory diseases within the mouth, and by the oral microbiome [12]. Numerous studies have investigated the association between periodontal diseases and oral cancers. A major challenge in reviewing observational studies that have reported associations between “periodontitis” and any cancer is the inconsistency in definitions of what constitutes periodontal disease [13,14]. Some studies have used objective measures, such as periodontal attachment levels, expressed sometimes as both severity and extent, radiographically assessed alveolar bone loss, or by combining both measurements [15]. Others have utilized self-reported measures, such as frequent gingival bleeding, or simplistic investigator measures, such as clinically evident gingival recession or bleeding on probing. The situation is further confounded by inconsistencies in the number of teeth quantified and the threshold values utilized [15]. Some studies have recorded tooth loss, but there can be no assumption that this reflects past periodontal disease: In spite of simplistic generalizations, such as that from the National Institute of Dental Research in the USA, which states that “periodontal disease” [undefined] is the major cause of tooth loss in adults [16].

To critically analyze the evidence of any links between “periodontal disease” and head and neck cancer, we reviewed the available literature on periodontal disease and HNC risk. Meta-analysis was performed according to the Preferred Reporting Items for Systematic Reviews and Meta-Analyses (PRISMA) guidelines to obtain an accurate assessment on the association between this important ‘oral health indicator’ and HNC.

## 2. Results

### 2.1. Study Selection

**Figure 1:**
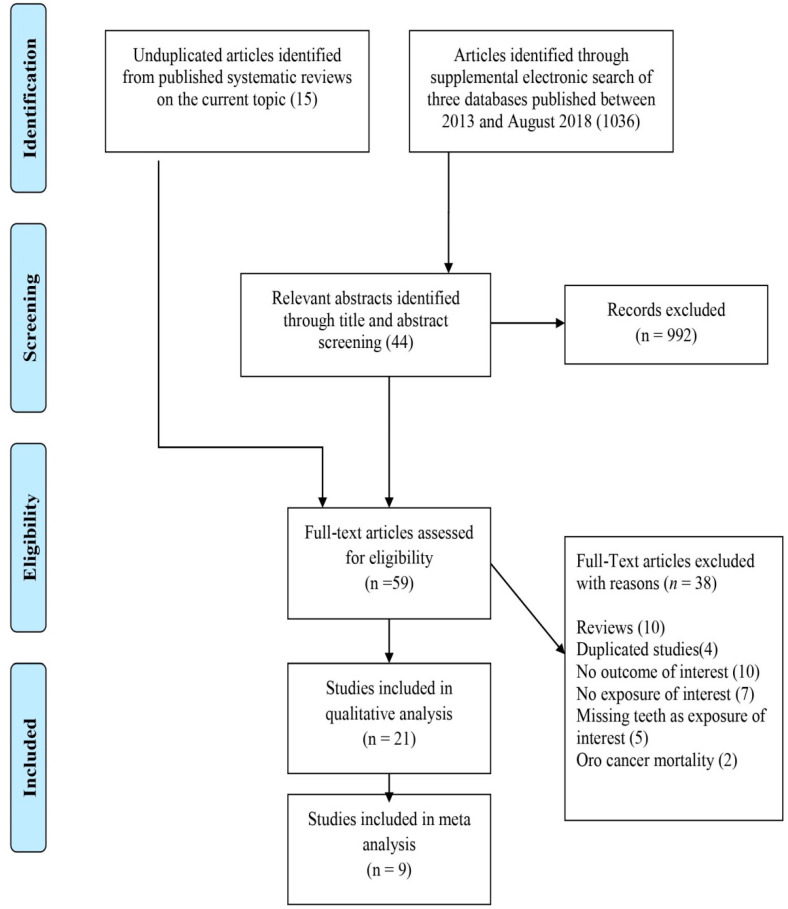
PRISMA flow diagram for systematic review depicting phases of identification of studies.

Nine articles were retrieved from two meta-analysis [17,18] and one systematic review [19] on the association of periodontal disease with HNC. After the review of 1036 abstracts through the supplemental search, an additional 9 case-control and 3 cohort studies with relevant outcomes were selected. Thus, overall, 21 eligible studies were identified after the review of 1051 articles, consisting of 4 cohort studies [20,21,22,23] and 17 case control studies [24,25,26,27,28,29,30,31,32,33,34,35,36,37,38,39] to be included in the qualitative analysis. A total of nine articles were eligible for inclusion in the meta-analysis. A detailed flowchart of the selection process is shown in Figure 1.

### 2.2. Characteristics of the Included Studies

#### 2.2.1. Case Control Studies

The main characteristics of the selected studies are illustrated in Table 1. Seventeen case-control studies reported a positive association between periodontal diseases and any HNC, with ORs over a wide range from 1.15 (0.81–1.63) to 9.33 (3.60–24.17), thus varying considerably in the strength of association. The selected studies were carried out in nations across Asia, America, and Europe, with the number of participants ranging from 75 to 3956. All studies had participants of both sexes, and among them, one study evaluated the risk for females and males separately [37]. Studies reported in earlier years had smaller sample sizes, whereas more recent data from multicenter studies [28,34,40] had a higher number of subjects for their analysis. The Alcohol-related Cancers and Genetic Susceptibility in Europe (ARCAGE) multicenter study had data collected over 13 centers from 9 European countries [34], whereas the International Head and Neck Cancer Epidemiology Consortium (INHANCE) reported pooled data from 13 case control studies [28].

#### 2.2.2. Cohort Studies

Positive associations between periodontal disease and head and neck cancer risk were reported in four cohort studies (Table 2). Three studies were conducted in the USA and one in Taiwan. Two studies enrolled males only [22,23] whereas one study focused entirely on females [21]. When cohort studies were considered exclusively, the studies reported similar associations with HR, ranging from 1.10 (0.64–1.87) to 2.25 (1.30–3.90). The cohorts were followed up for a period ranging from 5 years to 26 years allowing for the possible development of HNC. In a prospective study of 48,375 men from the USA, Michaud et al reported an HR of 1.15 (0.73–1.81) after adjusting for smoking [23]. In a subsequent paper on 19,933 men who were never smokers, they reported an elevated risk for esophageal and head and neck cancers, with an HR of 2.25 (1.30–3.90) [22].

### 2.3. Periodontal Disease Measurements

In total, 12 out of 21 articles used self-reported signs of gingival/periodontal disease, including self-reported gum bleeding [32,33,34,35,37], tooth mobility [25], self-reported diagnosis of periodontitis based on questionnaires [21,22,23], and “poor mouth condition” [28]. Objective measurements of periodontal disease were performed in only nine studies, utilizing a variety of measurements, including alveolar bone loss (ABL) [26,29,30,31,39], the Community Periodontal Index of Treatment Needs (CPITN) [36], visible assessments of clinical attachment loss [24], and ‘clinical diagnosis of periodontitis’, undefined [20,27]. 

### 2.4. Data Adjustment for Confounding Factors

All the included studies had adjusted for various confounding factors except the study by Rezende et al., which did not report any adjustments [36]. Moreover, among the 21 included studies that investigated this association, all studies except three [20,25,36] had reported their odds/hazard ratios after adjustment for the most important confounding factors for HNC, namely tobacco smoking and heavy consumption of alcohol. Amongst all studies from South and South East Asia, only one study, which was conducted on an Indian population, reported their odds ratio after adjustment for smokeless tobacco [24] even though the prevalence of smokeless tobacco use is high in South Asia [40,41].

### 2.5. Quality of Studies

A summary of the quality assessment of the studies according to the New-Castle Ottawa Scale is presented in Supplementary Table S1. Most of the case control studies were classified as fair. The main reason for downgrading the articles on the basis of the considered parameters from good to fair was due to the inadequacy of case definition and also a lack of community controls. All the cohort studies were of good quality.

### 2.6. Meta-Analysis

**Figure 2:**
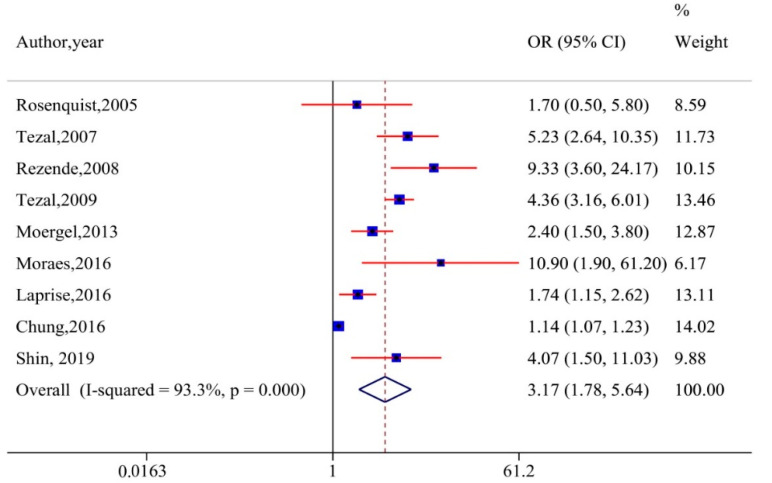
Forest plot of periodontal disease and risk of head and neck cancer (random-effects model). CI, confidence interval.

Twenty-one studies evaluating the relationship between periodontitis and head and neck cancer were included in our qualitative analysis. However, meta-analysis was performed by pooling the OR/RRs from nine articles that measured periodontal diseases with valid instruments. The combined effect estimate (pooled OR) using the random-effects model is presented in Figure 2. The overall results suggested a threefold increase in the risk of HNC in populations with periodontal diseases [OR 3.17, 95% CI, 1.78–5.64]. However, substantial heterogeneity was detected across the included studies (I^2^ = 93.3%). 

### 2.7. Sub-Group Analysis and Sensitivity Analysis

**Figure 3:**
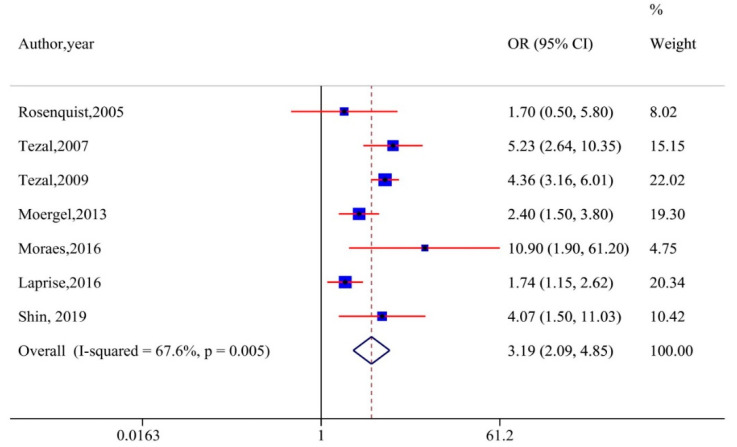
Sensitivity analysis plot excluding studies that did not adjust for smoking and alcohol.

**Figure 4:**
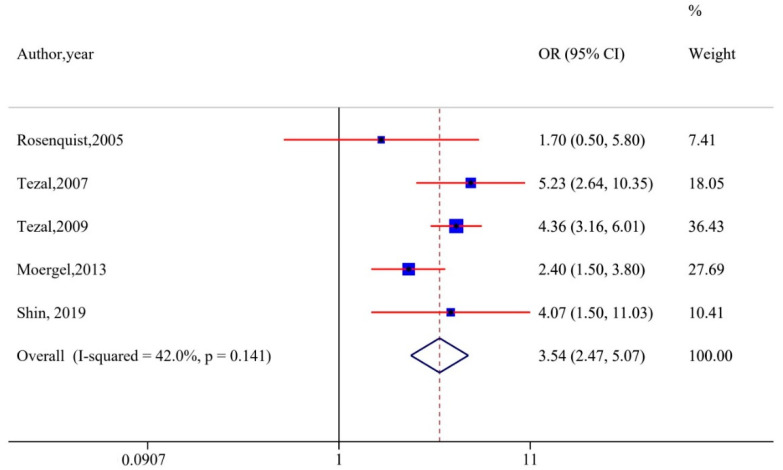
Subgroup analyses based on alveolar bone loss.

Sensitivity analyses were performed by excluding studies without adjustments for smoking and alcohol. The analyses demonstrated that the results remained robust (OR, 3.19 [95% CI, 2.09–4.85], I^2^ = 67.6%) (Figure 3). Of the nine studies included in the meta-analysis, five reported ABL values as measures for periodontitis. Hence, we performed a sub-group analysis with these: A significant association remained (OR, 3.54 [95% CI, 2.47–5.07], I^2^ = 42%), with a decline in heterogeneity (Figure 4). Tezal et al. reported a 5.23-fold increase in risk of cancer of the tongue with every millimeter of bone loss measured on a radiographic image utilizing a millimetric grid to convert image measurements from pixel to millimeter [35].

### 2.8. Publication Bias

The effect estimate (OR [3.17, 95% CI, 1.78–5.64]) was derived from the primary meta-analysis, which consisted of nine studies. However, the funnel plot looked asymmetric with evident bias in publications (Figure S1) and Egger’s test demonstrated the presence of small-study effects (Figure S2). 

## 3. Discussion

Interactions between periodontal diseases and systemic health are well established. However, the recent upsurge of publications relating inflammation and cancer, and the visible local inflammation in periodontal diseases has encouraged scientists to explore the relationship between periodontal disease and cancer risk. The overall findings from our qualitative and quantitative analyses assert an association between periodontal disease and HNC. However, the cross-sectional design utilized by most of the included studies limits affirmation of the course of this association, nevertheless revealing periodontal disease as a risk indicator for HNC. Furthermore, four large trials that evaluated the risk prospectively showed that people with periodontal disease had an increased risk of developing head and neck cancer. The most recent and comprehensive prospective study reported an elevated risk of HNC development, with HR of 2.25 (1.30–3.90) for periodontal disease alone and 6.29 (2.13–18.6) for periodontal disease with tooth loss (presumptively indicating a more advanced periodontitis) among males [22].

To date, two meta-analyses have been published investigating the relationship between periodontal disease and risk of HNC, the most recent in 2013 [17,18]. Although our results are consistent with these, the current review presents the most comprehensive summary of findings to date, by the addition of 11 recent studies for qualitative analysis and includes several large multicenter trials. Moreover, we utilized robust evidence synthesis means in our quantitative meta-analyses. We included studies that have objectively reported the presence of periodontitis with sound methods and excluded studies that accounted for periodontal disease by employing self-reported measures. Results from studies that investigated the reliability of the self-reported periodontal status in reflecting the actual clinical condition remain inconsistent [42,43]. Previous reviews have included these studies with self-reported measures in meta-analysis. Questionnaire evaluations have inherent limitations, which include recall and response biases, the latter involving social sensibilities [44]. Even though studies have previously tried to validate the findings from the questionnaires by clinical examination of a subset of the cases enrolled in the study, there are concerns regarding the reliability of self-reported periodontal outcomes assessed through questionnaires [13,44]. Nevertheless, the significance of findings from recent prospective studies that involve large numbers of participants using self-reported outcomes and validated questionnaires cannot be undermined [20,21] (Table 2).

One of the major challenges in assessing the relationship between periodontal disease and other diseases is the variability in measurement tools used to define disease. In the included studies, periodontal disease has been measured by self-reported outcomes of periodontitis or clinical measurements, such as attachment levels, pocket depths, and alveolar bone loss from panoramic radiographs [45]. Clinically, the diagnosis of periodontitis is based upon assessment of clinical attachment loss (CAL), probing depth (PD) measurements, evaluation of bleeding on probing (BOP), and evidence of loss of the alveolar bone, with or without radiography [46,47]. Even though standard definitions have been proposed [46,48], there is no uniformity in the methods used in the available studies [45,46,48,49]. Moreover, the number of teeth measured also varies between studies. Thus, the bias we encounter in our meta-analysis is inevitable [17,50,51,52,53,54,55,56,57]. For the future, internationally agreed standard definitions must be used [45,49].

Periodontitis and HNC share many factors in addition to heavy use of tobacco and alcohol, both environmental and inherited [58,59,60,61], and the exact interplay between shared features is yet to be determined. Thus, it could be argued that periodontal disease and HNC are both independent outcomes of a similar underlying genetic predisposition coupled with environmental risk factors. However, the majority of the studies included in our review reported a positive association between periodontal disease and HNC after controlling for these important confounding factors. Interestingly, sensitivity analyses with only those studies that adjusted for these factors did not influence the result, which points towards the fact that PD can be considered an independent risk factor for HNC (Figure 4). Moreover, the recent prospective study conducted on males who never smoked reported a significantly higher risk of HNSCC in periodontally compromised patients when compared to healthy males [22], indicating an independent risk factor. In deciphering the exact nature of the association, it is vital to determine whether treatment of periodontal disease could reduce HNC risk and improve prognosis. A study from Taiwan reported that the overall risk of developing cancer was significantly lower in patients who received periodontal treatment as compared to those without treatment [62]. However, we need more high-quality randomized controlled intervention trials to evaluate whether a reduction in risk can be achieved, so that maintenance of periodontal health could be incorporated as an integral part of public health policy. Along with regular tobacco and areca nut cessation campaigns, and dietary advice, sensible measures can be taken to educate individuals about the importance of oral health in the prevention of systemic diseases, including cancer.

Many studies have considered tooth loss as a marker for periodontal disease [13,63]. As discussed above, the end stage of periodontal disease is often loss of the supporting alveolar bone, leading to tooth loss. Hence, missing teeth are considered as a surrogate marker for periodontal diseases. The association between HNC and tooth loss was first investigated by Zheng et al in 1990, who identified this as a novel risk indicator for HNC [64]. Since then, numerous studies and reviews have investigated the association of tooth loss and risk of HNC [17,65]. However, the reasons for tooth loss vary from society to society, the most potent variable being access to dental professionals: In the absence of dental “care”, most people keep most of their teeth for most of their lives. Dental caries is a ubiquitous disease, and the major cause of tooth loss throughout life: For example, in China [66], India [67], and the USA [68]. In a study of Caucasian male health professionals, more than half of the males who reported to have only 0–16 remaining teeth did not report any history of periodontitis [23]. Moreover, the extent of tooth loss that might be considered as a threshold to ascertain the risk remains unclear [63]. Given that people lose teeth for reasons other than periodontitis, we consider tooth loss as a measure of overall oral health and hence excluded the articles in which tooth loss was only considered as a parameter to define periodontal disease.

Links between chronic inflammation and cancer are increasingly understood [12,69]. Periodontal diseases constantly contribute to systemic inflammation, elevating plasma levels of proinflammatory cytokines, acute phase proteins, and other proteinases [70,71,72]. The main hypothesis implicated on this association is oxidative damage to DNA [24]. In addition, recent studies suggest a direct role of periodontopathic bacteria in carcinogenesis. The two chief periodontal pathogens reported to be associated with HNSCC are *Porphyromonas gingivalis*, a member of the red complex consortium of Gram-negative anaerobes, and *Fusobacterium nucleatum* [7,73,74,75]. Infection by *F. nucleatum* has been shown to result in upregulation of several kinases involved in cell proliferation and DNA repair [76], whereas repeated exposure to strains of *P. gingivalis* has been shown to induce epithelial mesenchymal transition in vitro, which is a major event in carcinogenesis [77]. Moreover, P. gingivalis has also been shown to accelerate the progression of mammalian cells through the cell cycle via upregulation of cyclins and activation of cyclin-dependent kinases [73]. Chronic co-infection with *F. Nucleatum* and *P. gingivalis* has been shown to facilitate the progression of oral cancer in an in vivo model [78]. Both *P. gingivalis* and *F. nucleatum* have been linked recently to pancreatic and colorectal cancers, respectively [79,80]. Indeed, *Fusobacterium* has been labeled an “oncobacterium” in numerous cancers [81], and other bacteria found in association with periodontal disease have been associated with oral cancer in several isolated reports [82]. Apart from particular species, however, the current understanding is that it is the collective activity of consortia of microorganisms which drive cancer progression, and perhaps carcinogenesis itself, by their overall proinflammatory metabolites.

## 4. Limitations

Though our review covers several geographic regions and ethnic groups, most studies were performed in developed countries: The drivers in low and low-and middle-income countries might be different [83]. Smoking and alcohol consumption as possible confounding factors are clearly not eliminated though many authors have made efforts to adjust for them. Monitoring of smoking and alcohol consumption is challenging and only a well-conducted prospective study may be able to remove the confounding role of these two main risk factors for HNSCC. Although most studies were of fair quality, the combined effect estimate (OR) using the random-effects model revealed significant heterogeneity, attributable to the differences in population characteristics, sample sizes, study setting, and definitions of exposure. There is significant publication bias, as visualized in the Begg’s funnel plot, especially when less than 10 studies are included in a meta-analysis [41]. Asymmetry in the current funnel plot may be attributed to reporting bias: Statistically significant “positive” findings are more likely to achieve publication based on their effect estimates [54,84,85]. Studies with smaller sample sizes and less significant results are likely to be avoided, either by editors or the authors themselves when they lack interest for working more on such studies [84,85]. It was recently reported that half the funnel plots contained in Cochrane reviews exhibited some asymmetry, but in most cases, these biases did not affect the conclusions [86].

## 5. Materials and Methods

The methodology of this review was documented as a protocol and registered in PROSPERO (CRD42018074223).

### 5.1. Literature Search and Selection

The search strategy was defined based on the following PECO framework (P: population, E: exposure, C: comparator, O: outcome):

- P—Any population.
- E—Exposed to periodontal diseases.
- C—Not exposed to periodontal disease,
- O—Head and neck cancer,

The strategies for the screening and inclusion were decided by authors in advance. A thorough electronic search of literature for observational studies published to identify articles without date limitations, using specific search terms. The search was conducted on three major databases, PubMed, Scopus, and Embase, utilizing comprehensive literature search strategies with keywords that included “Head and Neck Neoplasms”, “Mouth Neoplasms”, “Tongue Neoplasms”, “Mandibular Neoplasms”, “Carcinoma, Squamous Cell”, “Oropharyngeal Neoplasms”, “Facial Neoplasms”, “Pharyngeal Neoplasms”, “Laryngeal Neoplasms”, “Periodontal Diseases”, “Periodontitis”, “Periodontal Attachment Loss”, “Periodontal Pocket” “Alveolar Bone Loss”, “Tooth mobility”, and “loose teeth”. Boolean operators, such as "and" and "or", were used to stratify the search. Moreover, manual searches of reference lists of relevant reviews were also conducted in order to exclude the possibility of omitting any important study. The search strategy was confined to English language comprising human studies. 

### 5.2. Eligibility Criteria

Two authors independently evaluated the eligibility of all the retrieved studies. The level of agreement between the authors was calculated utilizing Cohen’s kappa coefficient at every level of selection. To improve the sensitivity, papers were excluded only if both authors eliminated them based on the title and abstract and disagreements were resolved by discussion with a third author. 

The selection was done according the following criteria:

Observational studies (both case control and cohort) that assessed the association between any measure of periodontal status with HNC were included if they met the following criteria: (1) Controls and cases were clearly defined and (2) hazard ratios (HRs), odds ratios (ORs), or risk ratios (unadjusted/adjusted) and related 95% confidence intervals (CIs) were reported, or else the figures that can help to determine any of these ratios were present in the article.

Head and neck cancer (HNC) in our study was defined as any malignant neoplasm of the lip and oral cavity, any part of the pharynx, larynx, sinuses, and salivary glands reported collectively or individually. ‘Periodontal disease’ was defined as the presence of gingivitis and/or periodontitis by employing objective methods (clinical or radiographic assessment) and subjective methods (self-reported based on questionnaires or face-to-face interviews). Similar definitions were described in other reviews on the association of periodontal diseases and systemic conditions [87,88] Measures considered for clinical assessment of gingivitis included bleeding on probing and use of one of the gingival indices widely accepted in the specialist literature [89]. Measures considered for clinical assessment of periodontitis included clinical attachment loss, alveolar bone loss (ABL), measures of the depth of periodontal pockets, measures of attachment loss, records of tooth mobility, and various periodontal indices.

### 5.3. Data Extraction

Screening of the titles and abstracts was performed by two authors (DG and RKM) and relevant information from the full texts selected were extracted and tabulated. Two authors independently extracted data regarding the characteristics of each study using a standardized data collection form. When involving more than a single study involving the same sample group, the report with the most recent and comprehensive data on the study population was used.

### 5.4. Quality of Studies

Quality assessment of each of the included studies was performed using the Newcastle–Ottawa scale (NOS) for case-control and cohort studies [90]. The studies were assessed based on three separate parameters (selection, comparability, exposure) and received values ranging from 0 to 9 stars. Studies were classified as poor (<5), fair (5–7), and good (>7) based on the scores received.

Two authors independently graded the quality of each article and no discrepancies were identified.

### 5.5. Data Analysis

A meta-analysis using the random-effects model was performed to estimate the pooled odds ratio (OR) with a 95% confidence interval (CI) of studies that used objective measures to describe periodontal diseases. In the studies in which the hazard ratio (HR) or risk ratio (RR) were reported, we calculated OR through the following method. We directly considered HR as RR, and then transformed RR into OR by using the Zhang equation: RR = OR/(1 − P_0_) + (Pa × OR), where P_0_ refers to the risk of outcome of interest in the non-exposed group [91]. Heterogeneity was quantified using I^2^ statistics. Sensitivity analyses were performed to test the robustness of the results by excluding studies that reported unadjusted OR. Subgroup analyses were conducted based on alveolar bone loss as a measure of periodontal disease. Publication bias was assessed with a funnel plot and Egger regression asymmetry test [92] (*p <* 0.05 was considered to be statistically significant). This method, a rank-based data-imputation technique, considers the possibility of hypothetical “missing” studies that might be present, inputs their potential effects, and recalculates the effect size. For statistical analysis and graph generation, we used Stata version 15.0 (StataCorp, College Station, TX, USA).

## 6. Conclusions

This comprehensive systematic review and meta-analysis strongly supports that periodontal disease is an independent risk factor for head and neck cancer. Periodontally compromised individuals with co-existing lifestyle risk factors should be encouraged to monitor and maintain periodontal health to minimize cancer risk. Dental/medical care providers should provide regular oral health and cancer screenings to detect cancer at an early stage, thus improving survival and quality of life. Standardized definitions and measurements of periodontal disease are necessary for future epidemiologic studies, which should include longitudinal studies of the oral and periodontal microbiome. Ultimately, this will allow discrimination of patients at high risk of cancer, in this era of personalized medicine.

## Figures and Tables

**Table 1:** Characteristics of selected case control studies in detail.

Author	Year	Design	Population	Cases	Controls	Age Range	PD Assessment	Subtype	OR (95% CI)
Talam-ini [35]	2000	case control	Italy	112	125	60 (median)	Self-reported gum bleeding	OSCC	2.4 (1.5–3.8)
Garrote [32]	2001	case control	Cuba	150	171	28–91	Self-reported gum bleeding	OC/OPC	2.03 (0.66–6.25)
Balaram [37]	2002	case control	India	307	291	56 (22–85) M	Self-reported gum bleeding	OC	2.83 (1.71–4.68)
Balaram [37]	2002	case control	India	248	290	58(18–87) F	Self-reported gum bleeding	OC	3.35 (1.82–6.15)
Rosenq-uist [31] ^#^	2005	case control	Sweden	132	320	Ca:33–87/ Co:33–89	Alveolar bone loss	O/OPSCC	1.7 (0.5–5.8)
Tezal [29] ^#^	2007	case control	USA	51	54	54.6 ± 15.9	Alveolar bone loss	Oral tumor	5.23 (2.64–10.35)
Rezende [36] ^#^	2008	case control	Brazil	50	50	>40	CPITN	O/OPSCC	9.33 (3.60–24.17)
Tezal [30] ^#^	2009	case control	USA	266	207	Ca:56.89 ± 11.73/ Co:54.00 ± 15.45	Alveolar bone loss	HNSCC	4.36 (3.16–6.01)
Moergel [26] ^#^	2013	case control	Germany	178	123	60 (mean)	Mean bone loss	OSCC	2.4 (1.5–3.8)
Chang [33]	2013	case control	Taiwan	317	296	Ca-54.6/ Co 53.1	Self-reported gum bleeding	HNSCC	3.15 (1.36–7.28)
Eliot [38]	2013	case control	USA	513	567	Nil	Self-reported periodontal disease	HNSCC	1.09 (1.02–1.16)
ARCA-GE [34]	2014	case control	Europe	1963	1993	Ca-59.8 ± 10.1/ Co-59.8 ± 11.8	Self-reported Gum bleeding	Aerodigestive tract	1.15 (0.81–1.63)
INHA-NCE [28]	2016	case control	Multi-centric	2672	2634	15–80	Self-reported Gum disease	HNSCC	1.98 (1.68, 2.35)
Moraes [27] ^#^	2016	case control	Brazil	35	40	Ca: 55.1 ± 8.4/ Co:55.4 ± 9.4	Severity of periodontal diseasemeasured by probe	O/OPC	10.9 (1.9–61.2)
Laprise [24] ^#^	2016	case control	India	306	328	Ca-60 ± 10.8/ Co-59.2 ± 11.3	Gingival recession	OSCC	1.74 (1.15–2.62)
Mazul [25]	2017	case control	USA	212	321	20–80	Self-reported tooth mobility	OC	1.58 (1.05–2.37)
Shin [39] ^#^	2019	case control	Korea	146	278	Ca:63.8/Co:64.4	Alveolar bone loss	OSCC	4.066 (1.499 to 11.026)

Abbreviations: OC—Oral Cancer, OSCC—Oral Squamous cell carcinoma, OPC—Oropharyngeal cancer, HNSCC—Head & Neck Squamous Cell Carcinoma, OPSCC—Oropharyngeal Squamous Cell Carcinoma; ARCAGE—Alcohol-related cancers and genetic susceptibility in Europe, INHANCE—International Head and Neck Cancer Epidemiology Consortium; ^#^ Included in meta-analysis.

**Table 2:** Characteristics of the selected cohort studies in detail.

Author	Year	Duration	Population	Cases	Controls	Age Range	PD Assessment	Sub Type	HR (95% CI)
Michaud [23]	2008	18	USA	7863	40,512	40–75	Self-reported periodontitis	OPC	1.15 (0.73–1.81)
Chung [20] ^#^	2016	5	Taiwan	40,140	40,140	54.1 ± 11.5	Diagnosis of chronic periodontitis	OC (ICD-9-CM code 140–149)	1.20 (1.09–1.33)
Michaud [22]	2016	26	USA	1768	17,554	40–75	Self-reported periodontitis	OPC	2.25 (1.30–3.90)
Nwizu [21]	2017		USA	17,103	48,766	68.3(Mean)	Self-reported periodontitis	Lip, oral cavity and pharynx	1.10 (0.64–1.87)

Abbreviations: OPC—Oropharyngeal cancer, HNSCC—Head and Neck Squamous Cell Carcinoma, OPSCC—Oropharyngeal Squamous Cell Carcinoma. ^#^ included in meta-Analysis.

## Supplementary Materials

The following are available online at https://www.mdpi.com/2072-6694/12/7/1893/s1: Figure S1: Funnel Plot. Table S1: Quality Assessment by Newcastle–Ottawa Scale for Case Control and Cohort studies. Figure S2: Egger’s test.
