# Supplementary material for: Periodontal Diseases as Putative Risk Factors for Head and Neck Cancer: Systematic Review and Meta-Analysis
Source: Cancers (Basel). 2020 Jul 14;12(7):1893. doi: 10.3390/cancers12071893 (PMC7409086; doi:10.3390/cancers12071893)
Supplement: Supplementary file 1 [file cancers-12-01893-s001.pdf]

cancers

Supplementary Materials: Periodontal Diseases as Putative Risk Factors for Head and Neck Cancer—Systematic Review and Meta-Analysis

Divya Gopinath, Rohit Kunnath Menon, Sajesh K Veettil, Michael George Botelho and Newell W Johnson

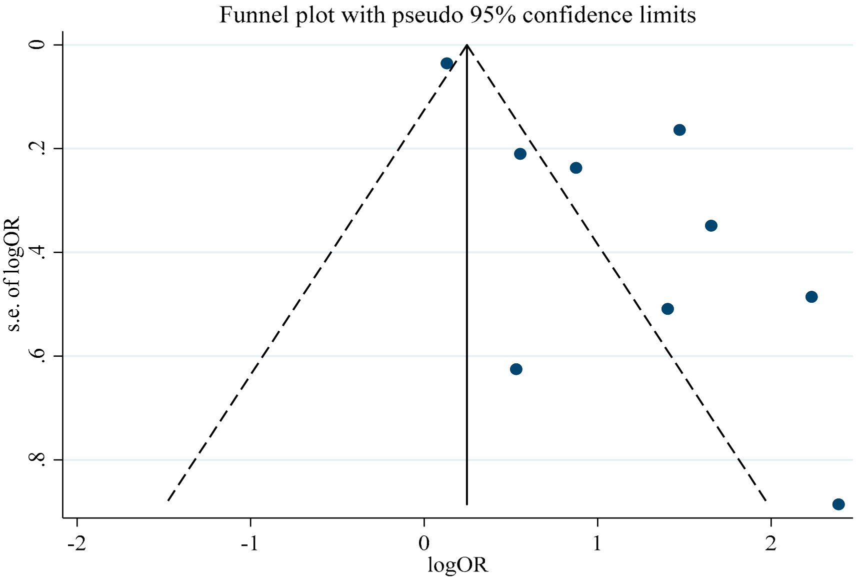

Figure S1. Funnel Plot.

Number of studies = 9 Root MSE = 2.399

Std_Eff	Coef.	Std. Err.	t	P>|t|	[95% Conf.	Interval]
slope	.0278582	.0996833	0.28	0.788	-.2078553	.2635718
bias	3.685492	.9907581	3.72	0.007	1.342721	6.028262

Test of H0: no small-study effects P = 0.007

Figure S2. Egger's test.

Table S1. Quality Assessment by New castle-Ottawa Scale for Case Control and Cohort studies.

Author	Year	Selection (max-4)	Comparability (max-2)	Outcome (max -3)	Total (max = 9)
		Case Control Studies			
Talamini	2000	***	*	**	6
Garrote	2001	**	*	**	5
Balaram	2002	**	**	**	6
Balaram	2002	**	**	**	6
Rosenquist	2005	**	*	**	5
Tezal	2007	***	*	**	6
Rezende	2008	***	*	**	6
Tezal	2009	***	*	**	6
Moergel	2013	***	*	***	7
Chang	2013	***	*	***	7
ARCAGE	2014	**	**	**	6
INHANCE	2016	**	*	***	6
Moraes	2016	**	*	***	6
Laprise	2016	***	**	***	8
Mazul	2017	**	*	***	6
Shin	2018	***	**	***	8
		Cohort studies			
Michaud	2008	****	**	**	8
Michaud	2016	****	**	**	8
Chung	2016	****	**	**	8
Nwizu	2017	****	**	**	8

© 2020 by the authors. Licensee MDPI, Basel, Switzerland. This article is an open access article distributed under the terms and conditions of the Creative Commons Attribution (CC BY) license (http://creativecommons.org/licenses/by/4.0/).
